# Potential sources of time lags in calibrating species distribution models

**DOI:** 10.1111/jbi.14726

**Published:** 2023-09-28

**Authors:** Franz Essl, Adrián García‐Rodríguez, Bernd Lenzner, Jake M. Alexander, César Capinha, Pierre Gaüzère, Antoine Guisan, Ingolf Kühn, Jonathan Lenoir, David M. Richardson, Sabine B. Rumpf, Jens‐Christian Svenning, Wilfried Thuiller, Damaris Zurell, Stefan Dullinger

**Affiliations:** ^1^ Division of BioInvasions, Global Change & Macroecology, Department of Botany and Biodiversity Research University of Vienna Vienna Austria; ^2^ Institute of Integrative Biology, ETH Zurich Zurich Switzerland; ^3^ Centre of Geographical Studies Institute of Geography and Spatial Planning, University of Lisbon Lisboa Portugal; ^4^ Associate Laboratory TERRA Lisbon Portugal; ^5^ Univ. Grenoble Alpes, Univ. Savoie Mont Blanc, CNRS LECA Grenoble F‐38000 France; ^6^ University of Lausanne Lausanne Switzerland; ^7^ Helmholtz Centre for Environmental Research – UFZ Halle Germany; ^8^ Martin Luther University Halle‐Wittenberg Halle Germany; ^9^ German Centre for Integrative Biodiversity Research (iDiv) Halle‐Jena‐Leipzig Leipzig Germany; ^10^ UMR CNRS 7058, Ecologie et Dynamique des Systèmes Anthropisés (EDYSAN) Université de Picardie Jules Verne Amiens France; ^11^ Department of Botany and Zoology, Centre for Invasion Biology Stellenbosch University Stellenbosch South Africa; ^12^ Department of Invasion Ecology Czech Academy of Sciences, Institute of Botany Průhonice Czech Republic; ^13^ Department of Environmental Sciences University of Basel Basel Switzerland; ^14^ Department of Biology, Center for Ecological Dynamics in a Novel Biosphere (ECONOVO) & Center for Biodiversity Dynamics in a Changing World (BIOCHANGE) Aarhus University Aarhus Denmark; ^15^ Institute for Biochemistry and Biology University of Potsdam Potsdam Germany; ^16^ Division of Biodiversity Dynamics and Conservation, Department of Botany and Biodiversity Research University of Vienna Vienna Austria

**Keywords:** climate change, climatic debt, colonization credit, extinction debt, invasion debt, mismatch, niche, projection, species distribution models

## Abstract

The Anthropocene is characterized by a rapid pace of environmental change and is causing a multitude of biotic responses, including those that affect the spatial distribution of species. Lagged responses are frequent and species distributions and assemblages are consequently pushed into a disequilibrium state. How the characteristics of environmental change—for example, gradual ‘press’ disturbances such as rising temperatures due to climate change versus infrequent ‘pulse’ disturbances such as extreme events—affect the magnitude of responses and the relaxation times of biota has been insufficiently explored. It is also not well understood how widely used approaches to assess or project the responses of species to changing environmental conditions can deal with time lags. It, therefore, remains unclear to what extent time lags in species distributions are accounted for in biodiversity assessments, scenarios and models; this has ramifications for policymaking and conservation science alike. This perspective piece reflects on lagged species responses to environmental change and discusses the potential consequences for species distribution models (SDMs), the tools of choice in biodiversity modelling. We suggest ways to better account for time lags in calibrating these models and to reduce their leverage effects in projections for improved biodiversity science and policy.

## 1 TIME LAGS, SPECIES DISTRIBUTION MODELS AND THE ANTHROPOCENE

Humans have always affected the environments they occupy. However, only in the last century have human‐induced modifications of the biophysical environment become pervasive, and they are still intensifying. Three‐quarters of terrestrial land and two‐thirds of oceans are moderately or strongly modified by humans (Ellis et al., 2010; Williams et al., 2020), the status of 47% of natural ecosystems has declined compared to their natural states (Díaz et al., 2019), the turnover time of biomass in terrestrial ecosystems has halved globally (Erb et al., 2016), and global average temperature has risen by c. 1.2°C above pre‐industrial levels (Pörtner et al., 2022).

These unprecedented rates and severity of anthropogenic pressures together with intentional and unintentional assisted movement of species worldwide have led to complex species and ecosystem responses. Yet, most biotic responses substantially lag behind immediate environmental changes, leading to so‐called disequilibrium dynamics (Svenning & Sandel, 2013). Range shifts of species tracking global environmental change became an increasingly frequent phenomenon in the 21st century (Antão et al., 2020; Chen et al., 2011; Essl et al., 2019; Lenoir et al., 2020), but many species do not keep up with climate warming, leading to climatic debts, even for mobile organisms like birds and butterflies (Devictor et al., 2012; Gaüzère & Devictor, 2021). While more than 37% of naturalized alien species were introduced into their new ranges only after 1970 (Seebens et al., 2017), many of these species are still in disequilibrium in their new range (Hui, 2023), not only due to dispersal limitations but also because of demographic lags, evolutionary lags, and lags affecting biotic interactions (e.g., Alexander et al., 2018; Crous et al., 2017; Early & Sax, 2014; Wagner et al., 2021). Consequently, c. 1 million species are predicted to be threatened with extinction (Díaz et al., 2019), meaning that their population and range sizes (i.e., extent of occurrence, area of occupancy) are shrinking. Depending on socio‐political decisions and the capacity for implementing and enforcing these, the velocity of environmental and biotic changes, caused, for instance, by climate (Loarie et al., 2009) and/or land use changes (Leclère et al., 2020), or the further introduction and spread of alien species (Seebens et al., 2021), is likely to even rise further in the future (IPBES, 2019).

Most anthropogenic pressures on biota have increased in recent years (IPBES, 2019), and new pressures with novel characteristics have emerged. Climate change, atmospheric nitrogen deposition (Staude et al., 2020; Stevens et al., 2018), pollution (e.g., noise, light pollution, plastic, pesticides; Halfwerk & Jerem, 2021; Sánchez‐Bayo & Wyckhuys, 2019) and ocean acidification (Hoegh‐Guldberg et al., 2007) are now acting everywhere, although with differing intensities and rates of change at different locations (e.g., Steffen et al., 2018; Wang‐Erlandsson et al., 2022). Depending on socio‐political decisions and the capacity for their implementation, the velocity of these environmental changes (Leclère et al., 2020; Loarie et al., 2009), biotic changes (Seebens et al., 2021) and species extinction rates (IPBES, 2019) are likely to increase further in the future. Anticipating and modelling species responses to new and increasingly changing environmental conditions altered by an increasing number of pressures is therefore becoming ever more challenging.

The most widely used approach to estimate and predict species' ranges is species distribution modelling (SDM), which relates species occurrence data (presence, presence–absence or abundance) to relevant environmental predictors (Guisan et al., 2017). Relying on the Grinnellian niche concept (Soberón, 2007), these approaches use statistical methods to estimate the realized environmental niche of species, which can then be used to predict the environmental suitability of species in space (i.e., to other parts of the globe) and time (i.e., under projected changes of environmental conditions) according to future scenarios (Elith & Leathwick, 2009; Thuiller et al., 2009).

Critical assumptions of SDMs are that species occurrence data cover the entire realized environmental niche of the selected species (Chevalier et al., 2022) and that species' occurrence data are in equilibrium (or at least nearly so) with the environmental predictors used in the models (i.e., the realized niche is filled and stable; Chevalier et al., 2022; Pearson & Dawson, 2003). While many inherent constraints of SDMs have already been widely discussed (e.g., Dormann et al., 2007; Guisan et al., 2017; Yates et al., 2018), the potential consequences of time lags in species occurrence and reporting for calibrating SDMs have not yet been comprehensively discussed. Here, we identify major sources of lagged species occurrence responses to environmental change and the ramifications they may cause for the reliability of SDMs. We discuss potential consequences for predicting species distributions, illustrate these phenomena using examples and provide suggestions for improved consideration of time lags in these models.

## 2 SPECIES RE‐DISTRIBUTIONS UNDER ENVIRONMENTAL CHANGE

Species occurrence data (i.e., presence‐only, presence–absence or, less frequently, abundance data) are quintessential for calibrating SDMs (Guisan et al., 2017). Species—or rather individuals and populations—respond to environmental changes in various ways. Responses can involve in situ adaptations (e.g., phenological, genetic, plastic or behavioural responses), or local extirpation and colonization, and spatial displacement, consequently affecting the occurrence, abundance and ultimately the geographic range of species. However, keeping pace with the current rate of environmental change is nearly impossible for most organisms (e.g., Devictor et al., 2012; Fricke et al., 2022; Pacheco‐Riaño et al., 2023; Svenning & Sandel, 2013). In particular, spatial redistributions of species require substantial time until a new spatial and environmental equilibrium is reached, and such distribution changes may take place at different velocities. Individuals and populations at the periphery of species' environmental and geographical ranges are at their ecological limit, and therefore, may have slower growth and reproductive rates, be less competitive (Svenning et al., 2014), and occur generally less frequently (Guisan et al., 2017). This potentially leads to more rapid occurrence changes within the original species range across short distances (e.g., colonization of newly suitable microsites, decline and ultimate extirpation at newly unsuitable microsites), but slower occurrence changes at species' range limits. Thus, the ecological processes underlying colonization of new geographic areas may happen at a different pace than extirpation at previously occupied sites, resulting in different lag times (Rumpf, Hülber, Wessely, et al., 2019; Rumpf, Hülber, Zimmermann, & Dullinger, 2019). For a species to go locally extinct, all individuals of a given population need to disappear, while a new habitat can be observed as colonized if only one individual is present. This potentially slower pace of extirpation can be amplified when populations still occur at a place that has become unsuitable, and thus is committed to extinction (extinction debt, e.g., Dullinger et al., 2013). For instance, adults of long‐lived species (e.g., trees) may occur outside their realized niche over extended periods after reproduction has ceased due to environmental change and form ‘Zombie Forests’ (Eriksson, 1996; Hill et al., 2023). Furthermore, if species are lost at fine scales (e.g., habitat level), but are still present at the landscape scale (e.g., raster cells of floristic mapping projects), losses may be masked in occurrence data commonly used for SDMs (Riva & Fahrig, 2023).

Adding to lags in species' responses to environmental change, recording species occurrences and integrating such data into large repositories also occurs with delays (Bailey et al., 2020). In particular, local losses of populations are particularly poorly recorded (Kuussaari et al., 2009), because the definite loss of a local population is often hard to ascertain, specifically for inconspicuous species, ephemeral species or species with long‐lived permanent stages (Downey & Richardson, 2016). By contrast, colonization of a new site is much easier to detect from monitoring and even opportunistic data. This implies that available distribution data from large repositories do not necessarily adequately represent the current distribution of species or their occurrence in current communities. This issue has been largely ignored in the literature so far.

## 3 ATTRIBUTES OF DISTURBANCES AND THE TEMPORAL NATURE OF SPECIES RESPONSES

**FIGURE 1:**
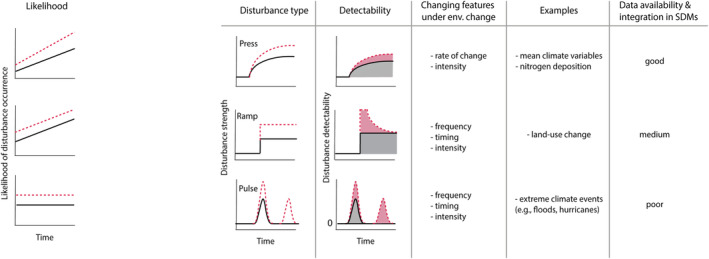
Different types of disturbances (i.e., press, ramp, pulse) under environmental change (black = before environmental change, red = the increase in disturbance strength and detectability after environmental change). Shown are detectability—that is, how difficult it is to observe and measure—different disturbance types over time; here, we are accounting for the shifting baseline syndrome (i.e., the gradual shift of reference points against a new state of a system is measured against) which is particularly relevant for slowly unfolding (press) and long‐lasting stable (ramp) pressures. Furthermore, we show examples for each disturbance type, data availability and integration in SDMs. For simplicity, changes in timing of disturbances are not shown. For a discussion on potential consequences for SDM calibration, see main text.

Environmental change may result in distinct types of disturbances (press, ramp, pulse), which can result in diverse biotic responses and lags (Inamine et al., 2022; Nimmo et al., 2015; Figure 1). Press disturbances are gradual changes in a system such as the mean states of climatic variables. They are observable all the time, can be measured relatively easily (although this may in practice be difficult due to fluctuations), and this is why they are commonly used in calibrating SDMs. Ramp disturbances are abrupt, instantaneous events that change the state of a system (e.g., changes in land use). These types of disturbances can only be observed for short periods, but their consequences may be evident for extended times (Dambrine et al., 2007; Polaina et al., 2019). In principle, precise data on their timing (e.g., when a patch of natural habitat has been converted to a field) are often lacking, especially for the future, and thus they are rarely considered in SDMs. Finally, pulse disturbances with subsequent recovery of the system to the original state (e.g., droughts, hurricanes, floods) typically occur more rarely and for (very) short time periods. They are rather easy to measure, but difficult to predict, and are also rarely used in SDMs. Importantly, and depending on the characteristics of the environmental factor, the affected ecosystem and the focal species, these different disturbance types are typically associated with different lag phenomena of species occurrences.

## 4 SOURCES OF TIME LAGS IN SDM CALIBRATION AND PREDICTION

SDMs rely on several fundamental assumptions (e.g., equilibrium of environment and species occurrence, unbiased recording, capturing of the full realized niche, all important niche descriptors included as predictors) on how species occurrences are spatio‐temporally represented and sampled (e.g., Elith & Leathwick, 2009; Guisan et al., 2017). These assumptions may become increasingly violated when species occurrences are responding dynamically to environmental changes and can translate into potential causes of calibration and prediction errors (Table 1).

**TABLE 1:** Sources for violating assumptions of species distribution models (SDMs) and potential consequences for time lags in SDMs.

Violation of SDM assumption	Potential consequences for time lags in SDMs	References
Occurrence (and sampling) data are in spatio‐temporal disequilibrium (i.e., environmental conditions are changing, or species is filling potential niche after recent introduction)	Occurrence data and predictor variables do not fully match, potentially not fully capturing realized species niche including time lags (cf. Figure 3)	Dormann et al. (2012)
Residuals of models are spatially autocorrelated	Dispersal limitation or lagged range expansion may mean that species occurrences do not fully represent realized species niche in equilibrium	Beale et al. (2010) and Dormann et al. (2007)
Realized niches are not conserved in space and time e.g., due to novel combinations of abiotic/biotic conditions (e.g., in areas where species have been introduced by humans or under novel environmental conditions after environment change in the historic range) allow species to occupy different realized niche spaces	Realized niches differ in size or in their location across environmental space in different parts of the species range or in different time periods	Alexander et al. (2016)
Mismatch between the temporal extent of predictor data used in SDMs and the date of observation of species	Occurrence data used may not fully reflect the environmental conditions as represented by explanatory data	Reside et al. (2010)
Full realized niche is not captured as a restricted environmental range is used to fit SDMs, resulting in a truncated niche. In particular, extremes of environmental conditions (i.e., pulse disturbances) are often underrepresented in occurrence data and thus have a higher uncertainty	Depending on the mistake in response curves caused by the truncation (e.g., sigmoid response instead of unimodal), SDM could predict persistence instead of extinction along part of the range. In particular, uncertainty at range margins may be higher, resulting in more unreliable predictions and time lags or even biased model results	Chevalier et al. (2022)

**FIGURE 2:**
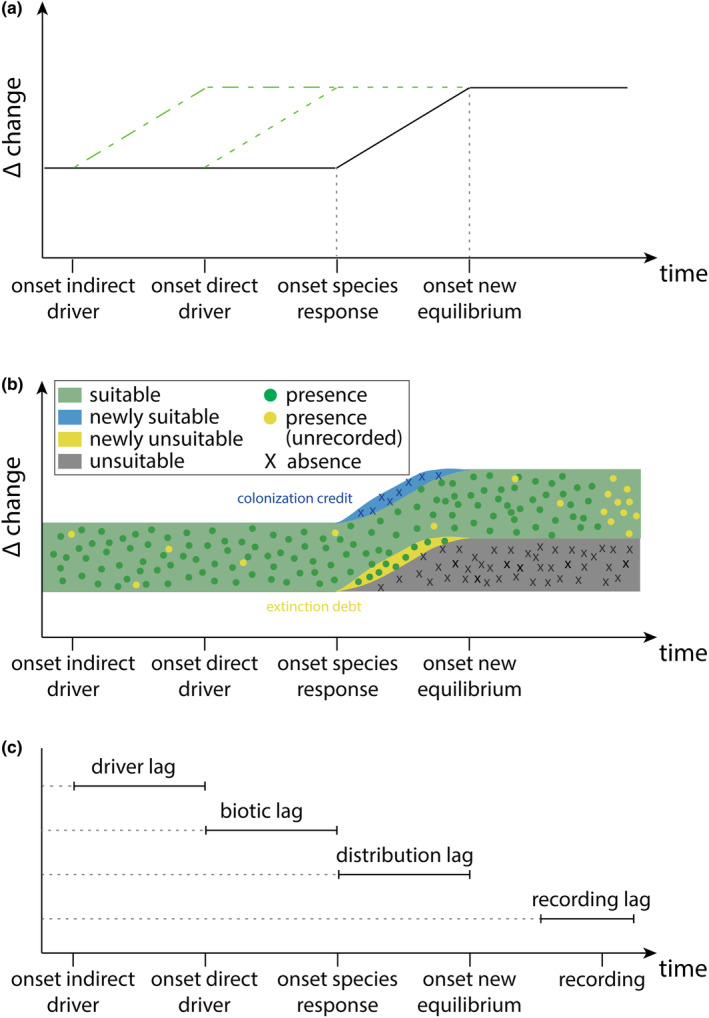
The sequence of temporal changes from indirect drivers to species occurrence responses (note that other lags are not shown) (a), the corresponding trajectory of change of species occurrences (b) and ensuing different elements of temporal lags (c). Note that a recording lag may even be more pronounced for SDMs than shown in (b), if occurrence data extend temporally over disturbance events (e.g., before and after the onset of climate warming). Unrecorded species' presences in (b) may occur at any time due to imperfect detection but tend to be more frequent for recently established populations (given uniform sampling intensity).

Environmental changes are precipitating cascading responses that accumulate across the reaction sequence of biotic responses from individuals to communities (Essl et al., 2015; Svenning & Sandel, 2013). For calibrating SDMs, this implies that changes in direct drivers (sensu Díaz et al., 2019), which are used as predictor variables in these models may lag behind changes of indirect drivers (= driver lag), are not yet fully translated into changes in species occurrences, implying that species distributions are not in equilibrium with environmental conditions (= distribution lag). This may be further exacerbated by a delay of human observations of species occurrences in the field, a process that we refer to as recording lag (Figure 2).

Widespread disequilibria of expanding, contracting and range‐shifting species due to rapidly changing environmental conditions imply that occurrence data of species increasingly reflect historic distributions in many cases and thus implicitly past environmental conditions. In fact, occurrence data are typically an amalgam of temporally structured data with a risk of truncation or bias of historic data (by lower sampling effort) and recent data (by recording lags). For instance, occurrence data on GBIF are strongly biased in terms of taxonomic, geographic and temporal coverage (Meyer, Jetz, et al., 2016; Meyer, Weigelt, et al., 2016). Furthermore, there are fewer than 500 million species occurrence records with observation dates before 2014 in GBIF, while the number of records with more recent observation dates is c. 2 billion (https://www.gbif.org/analytics/global). This has major implications for SDM parametrization, for instance when environmental predictors and distribution data do not fully match temporarily (Milanesi et al., 2020; Roubicek et al., 2010; Sanchez‐Martinez et al., 2021).

It is especially important to note that models may not be able to fully capture the realized niche of species under slowly unfolding press‐type environmental changes such as changes in mean annual temperature or precipitation sums, when species occurrence data used for calibrating SDMs are sampled from extended time periods (thus including data from before or during the environmental change), or (partly) from other periods than the environmental predictors used. However, it is not necessarily clear what the ideal temporal matching of time periods of occurrence data and environmental predictors is, as fingerprints of previous environmental changes may affect occurrence data sampled later. Thus, it may be appropriate to use environmental data preceding occurrence data used for model parametrization. The most appropriate approach will be contingent on species life histories and rates of change, and thus is highly context‐specific.

**FIGURE 3:**
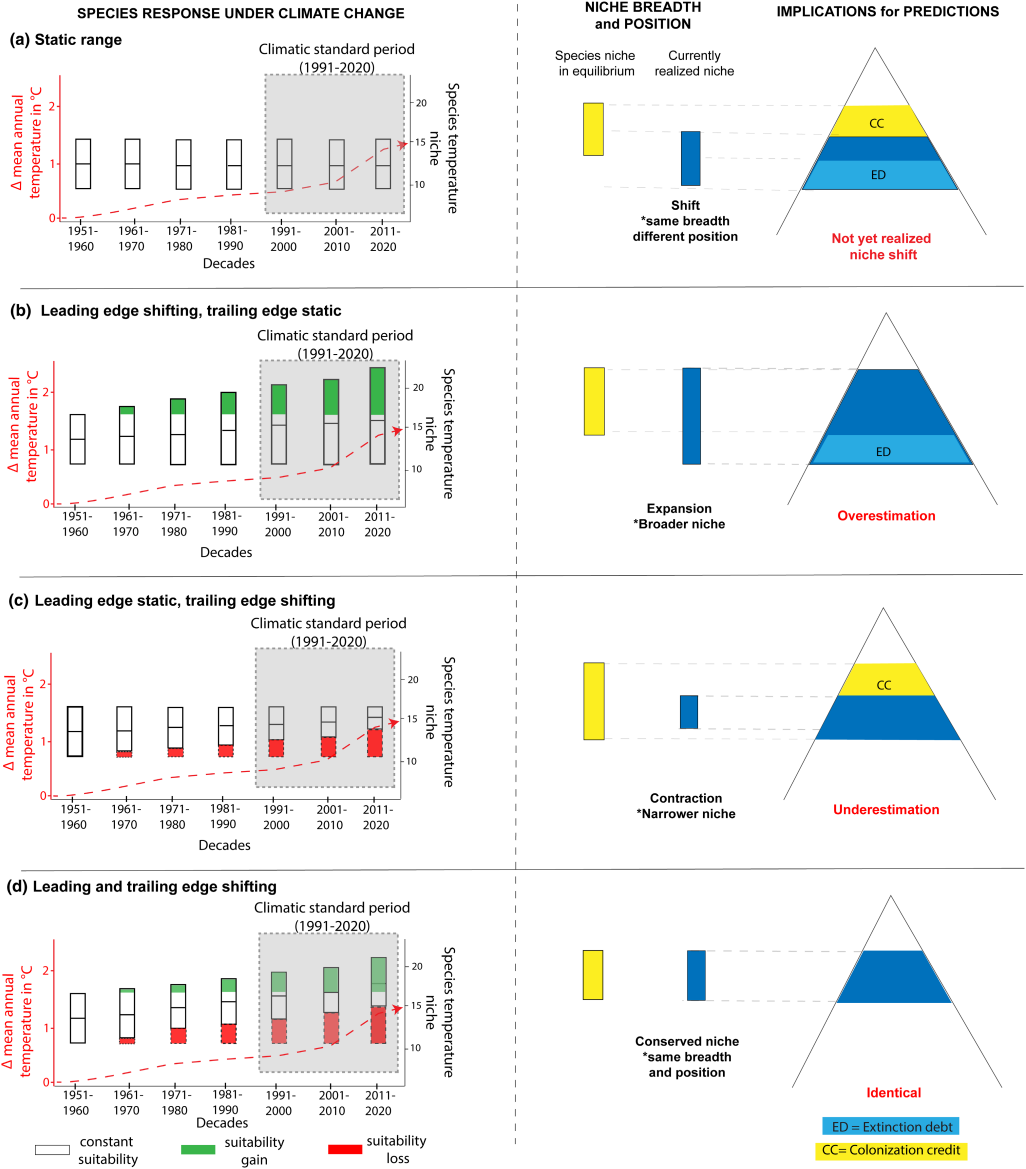
Sources for time lags in calibrating SDMs under changing climatic conditions and when species occurrence data are distributed over extended time periods or coming from other time periods than the climatic data. ‘Real’ = realized species niche when in equilibrium; ‘current’ = currently realized niche. Four different cases are shown: that is, species with static ranges in the time period of interest (a), species where the leading edge‐shifts, while the trailing edge remains static (b), species with static leading edge, while the trailing edge shifts (c) and species for which both leading and trailing edges are shifting (d). The climatic niche (here shown exemplarily for one variable, i.e., change in annual mean temperature) is shown here based on current climatic conditions (e.g., represented by the most recent climatic standard period, 1991–2020), calculated using current occurrence data versus historic distribution data before the onset of anthropogenic climate warming. The bars on the left represent decadal species temperature suitability, realized losses in niche space are shown in red, gains in green. The dashed red line indicates global warming over recent decades. On the right, the differences in size and position of calculated species' temperature suitability are shown, and the ensuing consequences for the spatial representation of suitable species niches are illustrated by using a stylized mountain.

For instance, depending on the response at the species' environmental or geographical limits (i.e., trailing and leading edges) which may be shifting or lagging behind environmental change, four different scenarios of how species niches are captured by SDMs can be identified (Figure 3). While lags at either the trailing or leading edge alone may cause over‐ or underestimations of realized species niches, equally delayed responses of both trailing and leading edge at the same time will not change the size of the calculated species niche, but will alter its location in environmental space. In theory, what would ultimately determine whether the issue biasing SDM calibration is the ratio between occurrences that truly reflect the niche of the species over the ones that are either novel or extirpated locations. Indeed, if most available data come from occurrence points that are at equilibrium, the calculated realized niche will be rather robust and, reciprocally, biases in the calculated realized niche can increase if many occurrence points come from populations at non‐equilibrium.

We summarized examples of time lags in SDM calibration from the literature to highlight that this phenomenon may indeed have important consequences for predictions (Table 2). We also provide suggestions on how these issues can be accounted for by careful data selection, changes in the methods used, or—at least—by interpreting results carefully. This will not, though, overcome the problem of species not (yet) being in equilibrium with their environment (i.e., a species that has not colonized all suitable environments), and hence, it is not possible to correct for this bias.

**TABLE 2:** Categories and examples of time lags in the calibration of species distribution models (SDMs). The corresponding causes (i.e., related to biological phenomena, or to being technical—related to data or modelling techniques), and the options for reducing time lags in SDMs. Key references are listed.

Domain	Phenomenon	Consequences for time lags in SDMs	Options for reducing time lags in SDMs	References
Biological	Compression of realized spatio‐temporal species niche due to human disturbance and habitat loss	Leads to niche truncation, i.e., incomplete realization of the potential niche (niche given non‐human biotic interactions)	Building integrated models that can use presence, abundance or biomass data from other sources, but also building models that can integrated theoretical or experimental responses of species to environmental predictors	Gilbert et al. (2022), Nüchel et al. (2018), Sales et al. (2022) and Talluto et al. (2016)
Biological	Dispersal limitation	Small‐ranged species are likely to generally have ranges severely constrained by large‐scale dispersal limitation, as has been shown for various groups e.g., trees; such species can be expected to often exhibit niche truncation	Integrate further data such as paleo‐records	Baselga et al. (2012), Seliger et al. (2021) and Svenning and Skov (2004)
Biological	Range expanding and alien species often have not colonized all suitable habitats or regions in the new range	Leads to the underestimation of the niche space, i.e., also a form of niche truncation and therefore of the full (potential) range	Include i) data from native range and all other areas the species has invaded, ii) use specifically tailored methods such as integrated species distribution models (iSDMs)	Hattab et al. (2017) and Srivastava et al. (2019)
Biological	Long‐living species that thrived under different climatic conditions, but still persist as ‘zombie species’, such as *Sequoia sempervirens* at its warm range limit in the western USA	Extreme case of mismatch between current environmental conditions and those at the time they germinated, grew and successfully reproduced	Using climatic (environmental data) from the appropriate time in history	Decker et al. (2021) and Hill et al. (2023)
Biological	Disequilibrium in biotic interactions in cases where range limits are not set directly by climate	Extinction lags, i.e., persistence in climatically ‘unsuitable’ sites until biotic interactions ‘catch up’	Develop trophic or competition species distribution models if the interactions and their directions are known	Alexander et al. (2018) and Leathwick and Austin (2001)
Biological	Sink dynamics, e.g., inbreeding depressions driven by human activity or environmental change.	Extinction lags, i.e., human‐mediated habitat change and fragmentation or emerging unsuitable environments sets in motion inbreeding depression, leading to population extinction at later stages.	Assessing demographic trends of populations and excluding (from modelling) those on a clear and consistent negative path?	Aguilar et al. (2019)
Technical	Retrospective validation methods used for estimating model's predictive ability may predict well future distributions, but poorly how distributions change	Forecasts of changes in spatio‐temporal occurrence may be inaccurate	Using change validation, i.e., using population change indicators over time and compare estimates of predictive performance, improves forecasts	Piirainen et al. (2023)
Technical	Time lags between realized species range changes and their recording, reporting and availability via repositories	Occurrence data used in the models may not represent the true (i.e., up to date) distribution of the species due to lags in recording/reporting	Explicitly consider these time lags, e.g., by using calibration time periods for which appropriate time has elapsed	Page et al. (2015), Smith and Blagoderov (2012) and Soltis et al. (2016)
Technical	Spatio‐temporal resolution of climatic data (too coarse spatial/temporal resolution of predictor values)	Species might be recorded as present in a given grid cell and allocated to coarse‐scale climatic conditions while they are only present in a microhabitat with deviating conditions (i.e., overly optimistic niche)	Using microclimatic data, especially in forest understoreys and mountains	Lembrechts et al. (2019)
Technical	Environmental predictors and species occurrences are sampled from aligned time periods	Species niches are often defined based on long‐term environmental averages spanning multiple decades (e.g., 30 years), but dates of species observation records vary within this timeframe. As a result, earlier records may reflect past suitable conditions, leading to inaccuracies in niche characterization	Calibrate SDMs having environmental predictors specified explicitly according to the date of each individual occurrence record	Dobson et al. (2023)
Technical	SDMs are typically parametrized with current distribution data	Initial models are calibrated on disequilibrium data that already include extinction debts and colonization credits	Using pre‐climate change distribution data	Rumpf, Hülber, Zimmermann, and Dullinger (2019) and Rumpf, Hülber, Wessely, et al., 2019)
Technical	Knock‐on effects of biotic interactions (e.g., extinction vortex)	Not taking into account biotic interactions such as hindered redistribution of one species can have effects on occurrences of interacting other species (e.g., delaying occurrence response under environmental change)	Explicitly incorporating interactions and indirect effects of environmental change into models reduces spatio‐temporal mismatch	Lucas et al. (2023) and Thuiller et al. (2018)

**FIGURE 4:**
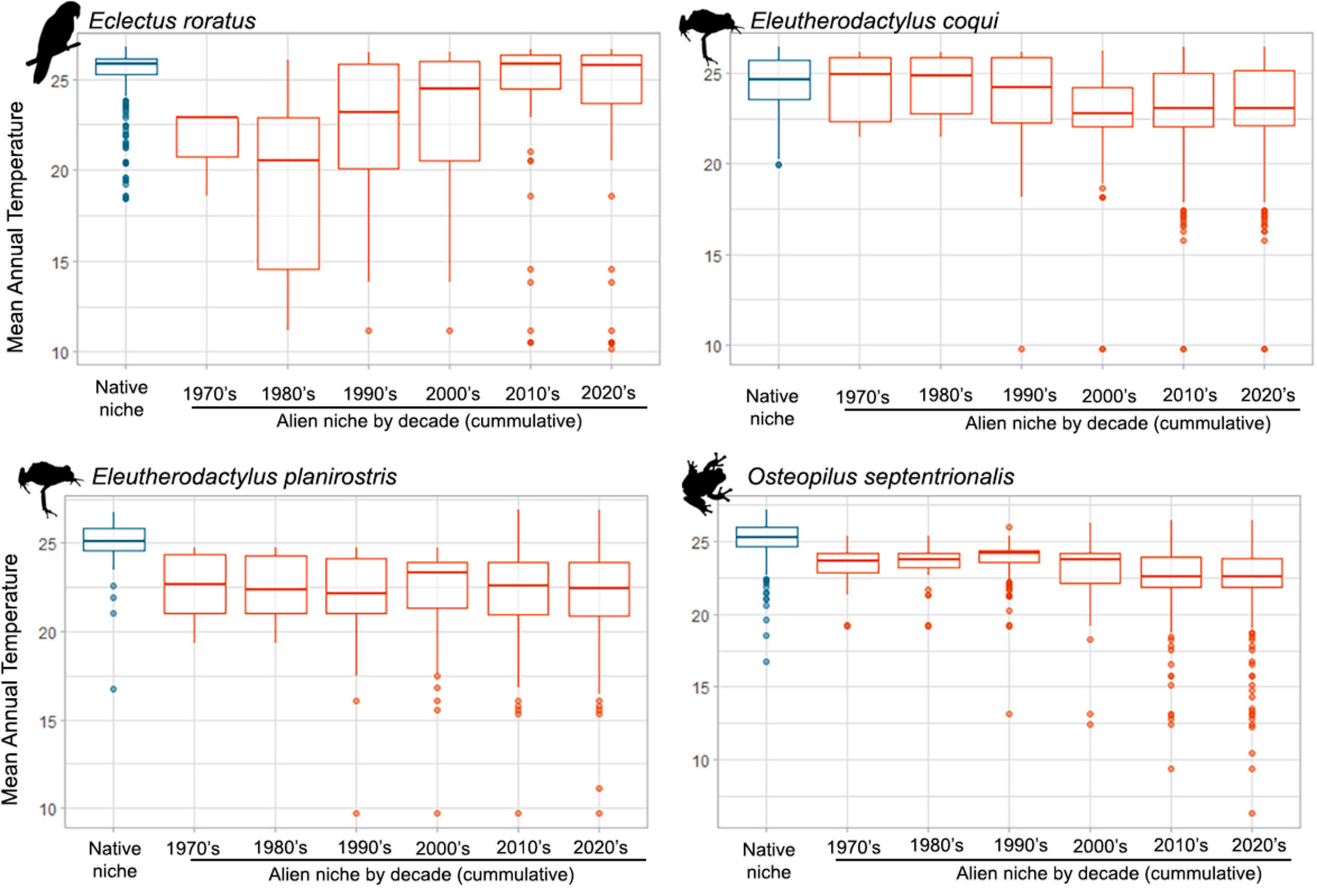
Realized thermal niches based on mean annual temperatures (at 30 s resolution, mean 1981–2010, taken from CHELSA 2.1, https://chelsa‐climate.org/downloads/) extracted for native and alien occurrences on GBIF of four island‐endemic vertebrate species with documented invasions in continental regions (data taken from García‐Rodríguez et al., 2023). In each plot, the blue box represents the species native temperature range. Orange boxes show the cumulative decadal change of mean annual temperature for occurrences in the alien range from 1970 onwards. For more details on data and approach used, see Data S1.

The spread phase of alien species colonizing new regions is often associated with niche shifts (e.g., Atwater & Barney, 2021; Guisan et al., 2014); it is likely that in the incipient spread phase, the realized niche in the invaded range is small and its location within the (larger) native species niche partly depends on chance (e.g., depending on the location of introduction), and that the realized niche expands during the spread process due to range and niche filling as invasion of additional habitats proceeds (Broennimann et al., 2014; Dullinger et al., 2009). Alien species that undergo boom‐bust dynamics may, however, experience a shrinkage of their realized niche in a latter phase of invasive spread (Strayer et al., 2017). As native ranges of island endemics are prone to niche truncation (Baselga et al., 2012), we explore this phenomenon by using four vertebrate species native to islands that have invaded continental regions (Figure 4). We found that indeed in the first spread phase the realized thermal niche is often distinct, yet not necessarily smaller, from the one observed in latter phases of spread. These exemplary insights highlight that temporal changes of the realized niche during the spread of alien species are indeed an important aspect to consider in SDMs. Conversely, the recent distribution of declining species may only represent a fraction of the potentially realized niche of the species (due to historic anthropogenic pressures or dispersal limitation after climate change; Sales et al., 2022; Scheele et al., 2017) and the remaining extant distribution may even be confined to suboptimal parts of the species niche. Particularly if the decline occurred a long time ago and the factors responsible for the decline have disappeared in the meantime (e.g., overhunting), current species occurrences may lag behind, particularly when re‐colonization of the former historic range is impeded by dispersal barriers and lack of available habitats (e.g., Cromsigt et al., 2012).

Ideally, the spatial resolution of environmental predictors used in SDMs should closely match the scale relevant for species occurrence (e.g., Elith & Leathwick, 2009; Guisan & Thuiller, 2005). In practice, however, scale mismatches due to lack of data or analytical constraints can be substantial (De Knegt et al., 2010) and may be in the order of several magnitudes, as the former typically are available at resolutions of kilometres, whereas for the latter the relevant scale usually is in the range of metres to tens of metres. In landscapes with high spatial heterogeneity such as mountains, environmental predictors like temperature, snow cover, soil depth and water retention may vary considerably over short distances (Scherrer & Körner, 2011). Particularly in these contexts, incorporating processes such as microclimate that are relevant for meta‐population dynamics directly into SDMs (Lembrechts et al., 2019) or training SDMs with environmental predictors that readily incorporate such processes (Lembrechts et al., 2019; Lenoir et al., 2017; Stickley & Fraterrigo, 2023) may help to capture time lag dynamics between the observed velocity at which species are shifting and the expected velocity that is needed to track the velocity at which isotherms are shifting according to coarse‐grained macroclimatic grids. Indeed, if SDMs were trained with the right microclimatic data at the right spatial resolution (Potter et al., 2013), as it is perceived by living organisms inside their habitats and not as it is measured by weather stations, then what we consider today as range‐shift dynamics lagging behind predictions or expectations from SDMs trained with macroclimatic data at coarse spatial resolutions could in fact just be attributable to a scale mismatch between environmental variables and the species‐relevant scale (Harwood et al., 2014). Accordingly, a recent study accounting for forest microclimate dynamics over time has demonstrated that observed changes in understorey plant community composition over time (i.e., community thermophilization) respond more closely to microclimate changes than to macroclimate changes across European forests (Zellweger et al., 2020). This suggests that the so‐called ‘climatic debt’ (Devictor et al., 2012) or changes in community composition that lag behind climate warming (Bertrand et al., 2011) could be related to scale issues—for example, the difference between macroclimatic and microclimatic debts.

## 5 SYNTHESIS

SDMs are designed for using data from a static environment when static species occurrences closely match environmental conditions, but they are widely used for predicting the consequences of environmental change on species distributions (i.e., dynamics). This mismatch between input data requirements and purpose of SDMs can cause biological, data‐related and methodological issues, which require specific attention, particularly as environmental change progresses. Lags in species redistributions which may be further exacerbated by reporting lags may affect the calibration of SDMs. We show that this in turn can have consequences for SDM predictions, as the potential environmental space for a species may be under‐ or over‐predicted, or shifted compared to predictions based on equilibrium distributions of the same species.

Work addressing temporal shortfalls in SDMs is underway on multiple fronts: substantial increases in species occurrence recording and quality (e.g., citizen science, online applications, eDNA, digital twin; de Koening et al., 2023) including structured data collection (e.g., biodiversity monitoring schemes), shortened recording lag times due to digital data collection and integration in large repositories, increasing open access to biological data and mobilization of historic occurrence data (e.g., museum specimens). Such advances are accompanied by the rapidly increasing availability of data relating to environmental change from a wide range of domains with ever increasing spatio‐temporal resolution and with near‐instant availability (e.g., due to Earth observation data), including novel data sets for hitherto neglected environmental change drivers. Finally, methodological advances in SDMs are constantly being made and become constantly easier to implement due to advances in computer power and available tools such as dedicated R packages.

While it is unfeasible to account for all time lag‐related aspects, we identify several ways to recognize and—whenever possible—account for possible temporal mismatches with species occurrences in SDM parametrizations (Table 1). We recommend that full disclosure of limitations of chosen modelling approaches and their implications on the results of lagged responses should be provided as a standard feature in SDM applications.

## AUTHOR CONTRIBUTIONS

FE conceived the presented ideas and led the writing. AG, BL and SD contributed to developing the initial ideas. All co‐authors discussed the initial ideas and contributed substantially to the writing.

## FUNDING INFORMATION

FE, SF, BL and AG appreciate funding by the Austrian Science Foundation FWF (grant no. I 5825‐B). DMR received support from the DSI‐NRF Centre of Excellence for Invasion Biology, Mobility 2020 project no. CZ.02.2.69/0.0/0.0/18_053/0017850 (Ministry of Education, Youth and Sports of the Czech Republic) and long‐term research development project RVO 67985939 (Czech Academy of Sciences). JCS considers this work a contribution to Center for Ecological Dynamics in a Novel Biosphere (ECONOVO), funded by Danish National Research Foundation (grant DNRF173) and his VILLUM Investigator project ‘Biodiversity Dynamics in a Changing World’, funded by VILLUM FONDEN (grant 16549). CC acknowledges support from the Portuguese Foundation for Science and Technology through funding to CEG/IGOT Research Unit (UIDB/00295/2020 and UIDP/00295/2020). DZ acknowledges funding from the German Science Foundation (DFG grant ZU 361/3‐1). WT from the European Union's Horizon Europe under grant agreement number 101060429 (project NaturaConnect). We are grateful to Michael Dawson and Richard James Ladle for soliciting this perspective piece, and we appreciate the helpful suggestions by Anna Cord.

## CONFLICT OF INTEREST STATEMENT

The authors declare to have no conflicts of interest.

## BIOSKETCH

**Franz Essl** is an ecologist with a research focus on biological invasions, climate change, macroecology and conservation science. He also has a keen interest in conceptual work aimed to improve the understanding of the foundations of biodiversity change in the Anthropocene.

## Supporting information


Data S1.

## Data Availability

The data used for producing Figure 4 are available upon request at the authors.
