# Supplementary material for: Potential sources of time lags in calibrating species distribution models
Source: J Biogeogr. 2023 Sep 28;51(1):89–102. doi: 10.1111/jbi.14726 (PMC10952696; doi:10.1111/jbi.14726)
Supplement: Supplementary file 1 — Data S1. [file JBI-51-89-s001.docx]

**Potential sources of time lags in calibrating species distribution models**

**Franz Essl^1,*^, Adrián García-Rodríguez^1^, Bernd Lenzner^1^, Jake M. Alexander^2^, César Capinha^3^, Pierre Gaüzère^4^, Antoine Guisan^5^, Ingolf Kühn^6,7,8^, Jonathan Lenoir^9^, David M Richardson^10,11^, Sabine B. Rumpf^12^, Jens-Christian Svenning^13^, Wilfried Thuiller^4^, Damaris Zurell^14^, Stefan Dullinger^15^**

^1^Division of BioInvasions, Global Change & Macroecology, Department of Botany and Biodiversity Research, University of Vienna, Rennweg 14, 1030 Vienna, Austria

^2^Institute of Integrative Biology, ETH Zurich, Universitätsstrasse 16, 8092 Zurich, Switzerland

^3^Centre of Geographical Studies, Institute of Geography and Spatial Planning, University of Lisbon, 1600-276 Lisboa, Portugal

^4^University Grenoble Alpes, University Savoie Mont Blanc, CNRS, LECA, Laboratoire d’Ecologie Alpine, F-38000 Grenoble, France

^5^University of Lausanne, 1015 Lausanne, Switzerland

^6^Helmholtz Centre for Environmental Research - UFZ, Theodor-Lieser-Str. 4, 06120 Halle, Germany

^7^Martin Luther University Halle-Wittenberg, Geobotany and Botanical Garden, 06099 Halle (Saale), Germany

^8^German Centre for Integrative Biodiversity Research (iDiv) Halle-Jena-Leipzig, Puschstrasse 4, 04103 Leipzig, Germany

^9^UMR CNRS 7058, Ecologie et Dynamique des Systèmes Anthropisés (EDYSAN), Université de Picardie Jules Verne, Amiens, France

^10^Centre for Invasion Biology, Department of Botany and Zoology, Stellenbosch University, Stellenbosch, South Africa

^11^Institute of Botany, Department of Invasion Ecology, Czech Academy of Sciences, CZ‐252 43 Průhonice, Czech Republic

^12^University of Basel, Department of Environmental Sciences, Bernoullistrasse 32, 4056 Basel, Switzerland

^13^Center for Ecological Dynamics in a Novel Biosphere (ECONOVO) & Center for Biodiversity Dynamics in a Changing World (BIOCHANGE), Department of Biology, Aarhus University, Ny Munkegade 114, DK-8000 Aarhus C, Denmark

^14^Institute for Biochemistry and Biology, University of Potsdam, Am Neuen Palais 10, D-14469 Potsdam, Germany

^15^Division of Biodiversity Dynamics and Conservation, Department of Botany and Biodiversity Research, University of Vienna, Rennweg 14, 1030 Vienna, Austria

* Corresponding author: Franz Essl, T: [43-1-4277-54372](tel:+43-1-4277-54372), F: 43-1-4277-9575; Email: [franz.essl@univie.ac.at](mailto:franz.essl@univie.ac.at)

**Supplementary Online Material 1**

**Supplementary Text S1**

To produce Figure 4, we first compiled occurrence data from the Global Biodiversity Information Facility (GBIF; [www.gbif.org](http://www.gbif.org)) for the full distributions of four island-endemic species that have invaded other regions of the globe. These species are a subset of a full dataset that was recently analyzed in another study (raw GBIF occurrences for the full dataset are available at <https://doi.org/10.15468/dl.b7zndx>) where we detected that their realized climatic niches frequently experience shifts in the alien range. From the compiled dataset, we classified as native those records overlapping with their respective species native range, based on range maps from IUCN ([www.iucnredlist.org](http://www.iucnredlist.org)) for amphibians (*Eleutherodactylus coqui, Eleutherodactylus planirostris, Osteopilus septentrionalis*, and upon request from Bird Life International ([http://datazone.birdlife.org/species/requestdis](http://datazone.birdlife.org/species/requestdis))) for birds (*Eclectus roratus*). Data cleaning and processing was conducted in R using the packages rgbif (Chamberlain & Boettiger, 2017), taxize (Chamberlain & Szöcs, 2013), CoordinateCleaner (Zizka et al., 2019) and raster (Hijmans & van Etten, 2010). We obtained alien records for these species from a database derived from the DASCO workflow (Seebens & Kaplan, 2022). DASCO combines geolocated occurrences of species available from GBIF with published regional checklists of alien species to identify alien occurrences more precisely.

We then extracted Mean Annual Air Temperature from the high-resolution CHELSA climatologies v2.1 for each occurrence from <https://chelsa-climate.org/>. The algorithm used to estimate temperatures in CHELSA is mainly based on statistical downscaling of atmospheric temperatures at a horizontal resolution of 30 arc sec (~ 1km) for the reference period 1979–2013 (Karger et al., 2017). With this data we constructed the realized native and cumulative decadal alien thermal niches for each of the species. The occurrence data includes information on collection date for most of the records (97 %).

**Supplementary References**

Chamberlain, S. A., & Boettiger, C. (2017). R Python, and Ruby clients for GBIF species occurrence data. *PeerJ Preprints*, *5*, e3304v1.

Chamberlain, S. A., & Szöcs, E. (2013). taxize: taxonomic search and retrieval in R. *F1000Research*, *2*, 191. https://doi.org/10.12688/f1000research.2-191.v1

Hijmans, R. J., & van Etten, J. (2010). raster: Geographic analysis and modeling with raster data. In *R package version* (Vol. 1).

Karger, D. N., Conrad, O., Böhner, J., Kawohl, T., Kreft, H., Soria-Auza, R. W., Zimmermann, N. E., Linder, H. P., & Kessler, M. (2017). Climatologies at high resolution for the earth’s land surface areas. *Scientific Data*, *4*, 1–20. https://doi.org/10.1038/sdata.2017.122

Seebens, H., & Kaplan, E. (2022). DASCO: A workflow to downscale alien species checklists using occurrence records and to re-allocate species distributions across realms. *NeoBiota*, *74*, 75–91. https://doi.org/10.3897/neobiota.74.81082

Zizka, A., Silvestro, D., Andermann, T., Azevedo, J., Duarte Ritter, C., Edler, D., Farooq, H., Herdean, A., Ariza, M., Scharn, R., Svantesson, S., Wengström, N., Zizka, V., & Antonelli, A. (2019). CoordinateCleaner: Standardized cleaning of occurrence records from biological collection databases. *Methods in Ecology and Evolution*, *10*(5), 744–751. https://doi.org/10.1111/2041-210X.13152
